# Online Service Function Chain Planning for Satellite–Ground Integrated Networks to Minimize End-to-End (E2E) Delay

**DOI:** 10.3390/s24227286

**Published:** 2024-11-14

**Authors:** Soohyeong Kim, Joohan Park, Jiseung Youn, Seyoung Ahn, Sunghyun Cho

**Affiliations:** 1 Major in Bio-Artificial Intelligence, Department of Computer Science and Engineering, Hanyang University, Ansan 15588, Republic of Korea; dreammusic23@hanyang.ac.kr (S.K.); yjs1104@hanyang.ac.kr (J.Y.); tpdud1014@hanyang.ac.kr (S.A.); 2 Spatial Wireless Networking Research Section, Electronics and Telecommunications Research Institute (ETRI), 218 Gajeong-ro, Yuseong-gu, Daejeon 34129, Republic of Korea; 1994pjh@etri.re.kr; 3 Department of Computer Science and Engineering, Hanyang University, Ansan 15588, Republic of Korea

**Keywords:** LEO satellite, online algorithm, routing algorithm, satellite–ground integrated network, service function chain

## Abstract

The combination of software-defined networking (SDN) and satellite–ground integrated networks (SGINs) is gaining attention as a key infrastructure for meeting the granular quality-of-service (QoS) demands of next-generation mobile communications. However, due to the unpredictable nature of end-user requests and the limited resource capacity of low Earth orbit (LEO) satellites, improper Virtual Network Function (VNF) deployment can lead to significant increases in end-to-end (E2E) delay. To address this challenge, we propose an online algorithm that jointly deploys VNFs and forms routing paths in an event-driven manner in response to end-user requests. The proposed algorithm selectively deploys only the essential VNFs required for each Service Function Chain (SFC), focusing on minimizing E2E delay—a critical QoS parameter. By defining a minimum-hop region (MHR) based on the geographic coordinates of the routing endpoints, we reduce the search space for candidate base stations, thereby designing paths that minimize propagation delays. VNFs are then deployed along these paths to further reduce E2E delay. Simulations demonstrate that the proposed algorithm closely approximates the global optimum, achieving up to 97% similarity in both E2E delay and CPU power consumption, with an average similarity of approximately 90%.

## 1. Introduction

Satellite–ground integrated networks (SGINs) are expected to play a key role in next-generation communication systems due to their ability to provide global and seamless services [1,2,3,4,5,6]. In the service scenarios envisioned for next-generation mobile communications, quality of service (QoS) requirements are becoming more granular and demand higher performance [7]. Moreover, next-generation communication systems aim to extend such requirements to global coverage, ensuring seamless service regardless of location [8,9]. While existing ground networks have made strides in meeting these QoS demands, they still suffer from coverage holes in remote areas such as sparsely populated regions, oceans, and volcanic zones. Recently, low-Earth orbit (LEO) satellites have garnered attention as a potential solution to this challenge, thanks to reductions in both launch and maintenance costs [10]. However, LEO satellites have limited computational capabilities, which may hinder their ability to meet the stringent QoS requirements of next-generation service scenarios [11]. Therefore, by combining the strengths of ground and satellite networks, SGIN can effectively address QoS demands while eliminating coverage gaps, positioning itself as a crucial paradigm for future communication systems.

Despite the emergence of SGIN, there are still unresolved challenges in providing next-generation services through SGIN. The hardware of existing LEO satellites is specialized for specific tasks [12,13]. In other words, each satellite can only perform predetermined tasks, leading to low resource utilization when these tasks are not requested. Upgrading hardware or adding new tasks requires launching additional satellites, resulting in high operating costs. Furthermore, integrating LEO networks with terrestrial networks is complex, as the technologies for each have evolved independently, necessitating new interoperability solutions [14].

Applying software-defined networking (SDN) and network function virtualization (NFV) technologies to SGIN offers a solution to these challenges [15]. SDN-based SGIN can enable more efficient service delivery through the use of Service Function Chains (SFCs) and Virtual Network Functions (VNFs). An SFC is an ordered sequence of network services that data traffic must traverse to meet specific requirements. These services are implemented as VNFs, which are virtualized network functions designed to provide flexible and efficient deployment. For example, an SFC might include a firewall (${\mathrm{VNF}}_{1}$), followed by a load balancer (${\mathrm{VNF}}_{2}$) and then an intrusion detection system (${\mathrm{VNF}}_{3}$). This structure allows the network to dynamically manage and optimize service delivery, reducing dependency on specialized hardware and improving flexibility and efficiency [12,13,14]. Consequently, SDN-based SGIN networks can deploy VNFs on demand at specific nodes, eliminating the need for additional hardware upgrades and enhancing resource utilization, ultimately enabling the provision of next-generation services without significant hardware investment.

### 1.1. Comprehensive Review of Recent Works

Various studies have been conducted on leveraging SDN in SGIN, particularly focusing on VNF deployment, SDN task offloading, resource allocation, orchestration, and multicast.

Numerous studies have been conducted on VNF placement with various objectives, ranging from minimizing end-to-end (E2E) delay to maximizing resource utilization. In SDN-based LEO networks, a VNF orchestration algorithm based on a time-evolving graph that considers the limitations of LEO satellites’ energy and computing resources was proposed in [13]. The authors of [16] suggested a distributed VNF deployment algorithm to minimize the E2E delay and transmission costs while deploying VNFs on LEO edge cloud satellites. A provisioning technique for dynamically determining the configuration of controllers based on control overhead and network load in SDN-based LEO satellite networks has been proposed [17]. The authors of [18] proposed a graph-attention-network-based algorithm for the smooth orchestration of Service Function Chains (SFCs) in LEO satellite networks for load balancing and QoS improvement. The authors of [19] presented an online VNF deployment algorithm based on deep reinforcement learning to maximize the resource utilization of LEO satellites for granular QoS and SFC requirements. In [20], an online algorithm was introduced, which chains VNFs in response to service requests from end users in an on-demand manner to minimize delays in LEO edge computing environments. The authors of [21] proposed a heuristic greedy algorithm to evenly maintain resource consumption among base stations in SGIN, deploy VNFs, and form routing paths for servicing SFCs. The authors of [22] proposed a greedy optimization algorithm to improve the link resource utilization of LEO satellites in SGIN by deploying VNFs and forming routing paths. To provide Internet-of-Vehicle services in SGIN combined with SDN, a Tabu search-based online algorithm for VNF deployment and scheduling to find sub-optimal results was proposed in [23]. The authors of [24] introduced a VNF deployment algorithm based on the stateless architecture of the core network and reliable context management to reduce the transmission frequency and service interruptions in SGIN. The authors of [12] proposed a time-expansion-based decoupled greedy algorithm to decrease the E2E delay while deploying VNFs and forming routing paths in SGIN.

Many studies have addressed topics in SDN-based SGIN, including task offloading and resource allocation. An algorithm for computational task allocation in LEO satellite edge computing was proposed to minimize the energy consumption of ground end users while satisfying QoS requirements [25]. A greedy task allocation algorithm for LEO satellite edge computing nodes was proposed to maximize computing resource utilization and minimize task-uploading costs [26]. The authors of [27] proposed a computation and communication resource allocation algorithm that enables task offloading in SGIN, thereby reducing delays while ensuring LEO satellites are energy-efficient. In [28], a channel-aware gradient fair association algorithm was proposed for collaborative offloading to achieve load balancing between LEO satellites. The authors of [29] designed an architecture for LEO edge-computing satellites supporting IoT devices and proposed a low-complexity offloading and scheduling algorithm. In [30], an online algorithm was introduced for resource allocation and offloading decisions to minimize power consumption for both end users and LEO satellites. In [31], a system model based on the Stackelberg game was designed for situations involving LEO satellite networks and large-scale end users, along with an offloading decision algorithm for end users based on the mean-field game.

Research has also been conducted on orchestration in SDN-based SGIN. In [32], three heuristic approaches were proposed for deploying VNFs on LEO satellites to improve the orchestration between SFCs. The authors of [33] proposed a potential game-based SFC delivery scheme to reduce orchestration and E2E delay among multiple SFCs installed in SGIN, thus optimizing SFC delivery. The authors of [34] proposed utilizing federated learning in SGIN to dynamically allocate resources to SFCs, satisfying the QoS requirements of end users. In [35], a reconfigurable SGIN architecture was designed, and resource allocation and orchestration algorithms were proposed for servicing SFCs.

There are a few studies related to multicast in SDN-based SGIN networks. The works of [36,37,38] proposed a software-defined multicast framework for improving Internet video delivery performance in SDN-based LEO satellite networks. The authors of [39] also proposed a routing algorithm that utilized unicast and multicast traffic engineering to enhance the performance of inter-satellite links in SDN-based LEO satellite networks.

### 1.2. Research Gap and Motivation

To date, various studies leveraging SDN in SGIN have been conducted, focusing on areas such as VNF placement, task offloading, and SFC orchestration. Among these, considerable attention has been given to VNF placement strategies to minimize E2E delay. However, two major technical challenges remain unresolved: (i) implementing VNF placement in an event-driven manner and (ii) jointly determining both VNF placement and routing paths. Firstly, service providers cannot predict when or what type of SFC requests will be made by end users [40]. One possible solution is to pre-deploy as many VNFs as possible on SGIN nodes to cope with this unpredictability; nevertheless, the limited storage capacity of satellites limits the number of VNFs that can be deployed. Even if a large number of VNFs were pre-deployed, the high traffic loads passing through these VNFs could quickly saturate the storage and computational capacity, resulting in a significant increase in E2E delay [41]. Therefore, a mechanism is needed to enable service providers to dynamically place VNFs in an event-driven manner as end-user service requests are made. Secondly, it is essential to jointly consider routing paths during VNF placement. SFCs consist of multiple VNFs distributed across various nodes. A simple shortest-path algorithm may overlook critical factors such as the available computational capacity and delays at specific nodes, which could ultimately lead to higher E2E delays. Thus, a solution is required that not only dynamically places VNFs but also optimizes routing paths to minimize E2E delays.

An online VNF deployment strategy aimed at minimizing E2E delay was proposed in [16,20]. While these works address the placement of SFCs, they do not optimize the corresponding routing paths. In [12], joint VNF deployment and E2E delay minimization strategies were introduced. However, the current research requires tens of seconds for algorithm operation, making it challenging to handle user requests in real time. To fill the research gaps, we propose an online algorithm that jointly forms routing paths and deploys VNFs in an event-driven manner.

### 1.3. Contributions

The main contributions of this study are as follows:

- We design a system model for VNF deployment and routing path formation in SGIN. Based on this model, we derive a mixed integer non-linear programming (MINLP) problem to minimize the sum of E2E delays for all end users, considering that the service provider does not know the type or timing of the SFCs requested by end users.
- We propose an algorithm to form a minimum hop region (MHR) to reduce the computational complexity of finding a global optimal solution for the presented MINLP problem. This algorithm identifies candidate base stations suitable for routing path formation, differentiating them from non-candidate stations.
- We propose an online algorithm that deploys VNFs and forms routing paths immediately in response to end users’ SFC calls. The algorithm first reduces propagation delay by forming routing paths and then minimizes the overall E2E delay by deploying VNFs on those paths. Our experimental results demonstrate that the proposed technique achieves 97% of the global optimal performance obtained through exhaustive search.

The remainder of this paper is structured as follows. Section 2 outlines the system model considered in this study. A detailed explanation of the proposed scheme is presented in Section 3, followed by the performance evaluation of the proposed algorithm in Section 4. Finally, the conclusions of this study are discussed in Section 5.

## 2. System Model

In this section, we describe the SGIN network model considered in this study. Subsequently, we explain the nodes and topology of the SGIN network. In addition, we model the characteristics of the SFCs and VNFs served through the SGIN network, along with the routing paths. Finally, we model the delay incurred when servicing SFCs from the service provider to the user through the routing paths formed.

### 2.1. Network Node Model

**Figure 1:**
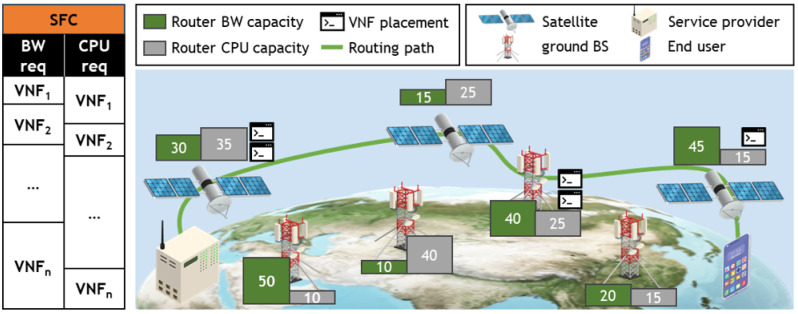
System model.

Figure 1 illustrates the SGIN network model considered in this study. The SGIN network contains four types of nodes: satellites, ground base stations (or routers), users, and service providers. In this paper, while the term “base station” is used, it can be interchangeably substituted with “router” in meaning. The set of service providers is denoted by SP and is assumed to be unique across the entire SGIN network. The set of satellites is represented by S and comprises $n_{s}$ satellites. Similarly, the sets of ground base stations and users are denoted by G and U, each comprising $n_{g}$ and $n_{u}$ nodes, respectively. The location of each node is described using two coordinate systems. One is the LLH coordinate system, which utilizes the latitude, longitude, and altitude, denoted by $\left(\lambda_{n},\theta_{n},h_{n}\right)$ for node *n*. The other is the ECEF coordinate system, centered at the Earth’s center, denoted as $\left(x_{n},y_{n},z_{n}\right)$ for node *n*. The distance between nodes *n* and *m* is defined as $r_{n,m}$. $r_{n,m}$ is calculated differently based on the node types *n* and *m*. If both *n* and *m* are ground-based nodes, the distance between them can be calculated using the Great-circle distance. Conversely, if one or both nodes are satellites, the distance between them is calculated using Euclidean distance. In summary, $r_{n,m}$ is defined as follows:

(1) $$\begin{array}{c} r_{n,m}=\left\{\begin{array}{c} E\cdot \tan^{-1}\left(\frac{\sqrt{H(n,m)}}{\sqrt{1-H(n,m)}}\right)\;\mathrm{if}\;n,m\notin S\\ \sqrt{{\left(x_{n}-x_{m}\right)}^{2}+{\left(y_{n}-y_{m}\right)}^{2}+{\left(z_{n}-z_{m}\right)}^{2}}\;o.w\; \end{array}\right. \end{array}$$

where *E* denotes the radius of the Earth, and H(n,m) is defined as

(2) $$\begin{array}{cc} H(n,m)= & \sin^{2}\left(\frac{\lambda_{n}-\lambda_{m}}{2}\right)+\cos\lambda_{n}\cdot \cos\lambda_{m}\cdot \sin^{2}\left(\frac{\theta_{n}-\theta_{m}}{2}\right) \end{array}$$

The communication link between nodes *n* and *m* is denoted by $l_{n,m}$. The set of all communication links is denoted by L, and the number of elements in L is ${\left(n_{s}+n_{g}+n_{u}+1\right)}^{2}$. We assume that a communication link is wired if both ends of the link are ground-based nodes and wireless if one or more ends are satellites. $r_{th}$ is the maximum communication distance required to form a communication link. If the distance $r_{n,m}$ between two specific nodes *n* and *m* is less than or equal to $r_{th}$, a communication link is assumed to exist between the two nodes. Otherwise, if the distance between the two nodes exceeds $r_{th}$, it is assumed that there is no communication link between them and direct communication is not possible. Thus, the communication link $l_{n,m}$ between the two nodes can be defined as follows:

(3) $$\begin{array}{c} l_{n,m}=\left\{\begin{array}{c} 1,\;\mathrm{if}\;r_{n,m}\leq r_{th},\\ 0,\;o.w, \end{array}\right.\;\forall n,m\in \mathrm{SP}\cup S\cup G\cup U. \end{array}$$

Finally, it is necessary to define the computing power of the base station nodes and the data rate of the communication links to model the computing and transmission delays. In SGIN, the role of a base station can be assumed by both ground base stations and satellites. The computing power of the base station node n∈S∪G is defined as $P_{n}$. In addition, the data rate of link $l_{n,m}$ is defined as $W_{n,m}$.

### 2.2. Service Function Chain Model

The set of SFCs provided by the service provider is denoted by C, where $n_{c}$ indicates the number of SFCs. Additionally, the set of VNFs is denoted by F, where $n_{f}$ is the number of VNFs. Moreover, $n_{n,f}$ represents the number of VNFs composing SFC *n*. Naturally, $n_{n,f}$ is a natural number less than or equal to $n_{f}$. Each VNF requires a certain amount of computing power for processing and generates a data size upon completion of the process. The computing power requirement of the VNF f∈F is defined as $p_{f}$, and the resulting data size after processing is defined as $w_{f}$.

The set of VNFs constituting SFC *m* is represented as $s_{m}=\left\{f_{m,1},f_{m,2},\ldots ,f_{m,n_{m,f}}\right\}$. The VNFs composing an SFC must be processed in a specific order. For convenience, SFC *m* is assumed to begin processing from $f_{m,1}$ and end processing with $f_{m,n_{m,f}}$. In this study, it is assumed that the VNFs composing an SFC are distinct from each other. However, the proposed algorithm can still operate even if the same VNF appears in different positions within the same SFC. When SFC *m* is invoked, the service provider must deploy all VNFs in $s_{m}$ at the base station located between the service provider and user. It is assumed that VNFs are not installed on the service provider or user. Variable $\alpha_{n,f}$ is a binary variable that indicates whether VNF *f* is deployed at base station node *n*. If VNF *f* is deployed at node *n*, $\alpha_{n,f}$ is set to one; otherwise, it is set to zero. If a node exists in which all VNFs composing SFC *m* are deployed, the routing path for processing SFC *m* is designed to traverse nodes $\alpha_{n,f_{m,1}}$ and $\alpha_{n,f_{m,2}}$ in sequential order starting from the service provider and ending at the user. In summary, the set of rendezvous points for routing paths to process SFC *m*, denoted by $A_{m}$, is defined as follows:

(4) $$\begin{array}{c} A_{m}=\{n|n\in S\cup G\;\mathrm{where}\;\alpha_{n,f}=1\;\forall f\in s_{m}\}, \end{array}$$

where $a_{m,i}$ indicates the *i*-th element of $A_{m}$. Here, the set of rendezvous points $A_{m}$ represents the nodes—either satellites (S) or ground (G) base stations—where the necessary VNFs are deployed to facilitate the SFC *m*. These nodes are crucial for effectively aggregating, routing, and processing data, ensuring that the service requirements for SFC *m* are met efficiently.

$A_{m}$ represents the set of rendezvous points for servicing SFC *m* but does not represent the actual routing path. Therefore, we define set B to denote the actual routing path for servicing SFC *m*. Specifically, $B_{m,i}$ is the set of communication links $l_{x,y}$ that represents the routing path from the node where $f_{m,i}$ is installed to the node where $f_{m,i+1}$ is installed. In other words, it represents the routing path from the rendezvous point $a_{m,i}$ to $a_{m,i+1}$ for servicing SFC *m*. If $f_{m,i}$ and $f_{m,i+1}$ are installed on the same node, that is, $a_{m,i}=a_{m,i+1}$, then $B_{m,i}$ is an empty set. If $l_{x,y}$ is an element of $B_{m,i}$, then $\beta_{x,y,m,i}$ is equal to one; otherwise, it is zero. $b_{m,i,j}$ is defined as the *j*-th element of $B_{m,i}$.

### 2.3. Communication Delay Model

In this study, the range of E2E delay for servicing SFC *m* is defined as the time taken for the traffic flow of SFC *m* to travel from the service provider to the end user. E2E delays include propagation, computation, and transmission delays. Propagation delays are determined by the distance of $l_{x,y}$ and vary based on whether the communication link is wired or wireless. The propagation delay $d_{l_{x,y}}^{p}$ for the communication link $l_{x,y}$ is defined as

(5) $$\begin{array}{c} d_{l_{x,y}}^{p}=\left\{\begin{array}{c} \frac{r_{x,y}}{c}\;\mathrm{if}\;l_{x,y}\;\text{is wireless link}\\ \frac{r_{x,y}\cdot \rho }{c}\;\mathrm{if}\;l_{x,y}\;\text{is wired link}\; \end{array}\right. \end{array}$$

*c* represents the speed of light and ρ denotes the refractive index of the wired link.

$d_{n}^{c}\left(f\right)$ is defined as the computing delay incurred when the base station node *n* processes the VNF *f*. The computing delay is determined by the computing power $P_{n}$ of node *n* and the computing requirement $p_{f}$ of VNF *f*. Specifically, the computing delay should increase inversely with the computing power of the node and directly with the computing requirements of the VNF. Additionally, in this study, it is assumed that if a base station installs multiple VNFs simultaneously, it allocates uniform computing resources to the installed VNFs. Considering these conditions, $d_{n}^{c}\left(f\right)$ can be defined as

(6) $$d_{n}^{c}\left(f\right)=\frac{\sum_{f\prime \in F}\alpha_{i,f\prime }\cdot p_{f}}{P_{n}}\;$$

$d_{l_{x,y}}^{t}\left(f\right)$ is defined as the transmission delay incurred when transmitting VNF *f*, whose processing has been completed through the communication link $l_{x,y}$. Similar to the computing delay, the transmission delay should increase inversely with the data rate of the link $W_{x,y}$ and directly with the data rate requirement of the VNF $w_{f}$. Additionally, if multiple VNFs need to be transmitted through the same communication link, the base station is assumed to distribute the bandwidth resources equally. Considering these factors, $d_{l_{x,y}}^{t}\left(f\right)$ can be defined as

(7) $$\begin{array}{c} d_{l_{x,y}}^{t}\left(f\right)=\frac{\sum_{m\in S}\sum_{i\in s_{m}}\beta_{x,y,m,i}\cdot w_{f}}{W_{x,y}} \end{array}$$

## 3. Proposed Algorithm

In this section, we define E2E delay using the delay functions defined earlier. In addition, we formulate a numerical problem of installing VNFs at base station nodes and forming routing paths to minimize the E2E delay of service provision through SFCs in SGIN. To solve this problem, we describe an algorithm for forming an MHR in Walker-delta environments. Finally, we introduce an algorithm for forming routing paths and determining the nodes for installing VNFs to service the SFCs in the formed MHR.

### 3.1. Problem Formulation

As mentioned previously, the scope of the E2E delay is defined as the traffic flow of the SFC from the service provider to the end user. The most natural form of E2E delay involves a sequential combination of computing, transmission, and propagation delays. For example, assuming servicing SFC *m*, first, transmission and propagation delays occur from the service provider to $a_{m,1}$. Second, a computing delay $d_{a_{m,1}}^{c}\left(f_{m,1}\right)$ occurs when processing the VNF at node $a_{m,1}$. Following processing of VNF $f_{m,1}$, the transmission delay $d_{l_{x,y}}^{t}\left(f_{m,1}\right)$ and propagation delay $d_{l_{x,y}}^{p}$ occur when transmitting traffic to $a_{m,2}$. This process is repeated until $a_{m,|s_{m}|}$ is used to compute the E2E delay. However, unlike computing delay, transmission and propagation delays have an additional factor to consider. Specifically, we must consider a scenario in which adjacent ordered VNFs within the SFC are installed on the same base station node. In this case, VNFs can be processed consecutively within a node without additional transmission or propagation processes, resulting in only a computing delay without transmission or propagation delays. Considering these conditions, the E2E delay function $D_{m}$ incurred when the service provider services SFC *m* can be defined as follows:

(8) $$\begin{array}{cc} D_{m}= & \sum_{a_{m,i}\in A_{m}}d_{a_{m,i}}^{c}\left(f_{m,i}\right)+\sum_{f\in s_{m}}\left(\min\left(1,|B_{m,f}|\right)\right)\cdot \sum_{j\in B_{m,f}}\left(d_{b_{m,f,j}}^{t}\left(f_{m,i}\right)+d_{b_{m,f,j}}^{p}\right)\; \end{array}$$

The first summation represents the computing delay. The middle summation checks whether adjacent VNFs within the SFC should be processed on the same node. |B| indicates the number of elements in B. If adjacent VNFs are installed at the same node, |B| becomes zero, indicating no transmission and propagation delay. Otherwise, the last summation enables the calculation of the transmission and propagation delays incurred by the actual routing path.

This study aims to minimize the E2E delay incurred when the service provider delivers all the SFCs by strategically deploying VNFs on base station nodes and forming routing paths for the SFCs. Accordingly, the following problem can be formulated.

(9) $$\begin{array}{cc} \min_{\alpha ,\beta } & \;\sum_{m\in C}D_{m} \end{array}$$

(10) $$\begin{array}{cc} \mathrm{subject}\;\mathrm{to} & \;\sum_{a\in A_{m}}\alpha_{m,f}=1,\;\forall f\in s_{m}, \end{array}$$

(11) $$\begin{array}{cc} \; & \;\sum_{i\in s_{m}}\sum_{l_{x,y}\in B_{m,i}}\beta_{x,y,m,i}=1,\;\forall l_{x,y}\in L, \end{array}$$

(12) $$\begin{array}{cc} \; & \;\alpha_{m,f}=\left\{0,1\right\},\;\forall f\in F,m\in C,\\ \; & \;\beta_{x,y,m,f}=\left\{0,1\right\}, \end{array}$$

(13) $$\begin{array}{cc} \; & \;\forall l_{x,y}\in L,f\in F,m\in C. \end{array}$$

The objective function (9) signifies that the E2E delay of all the SFCs provided by the service provider should be minimized. Constraint () implies that, within a single SFC, there should be at most one node where a VNF is installed. Importantly, this constraint does not mean that the same VNF cannot be installed on multiple nodes within the entire SGIN; rather, within the routing path for servicing a single SFC, only one node with that VNF should exist. The constraint () ensures that the traffic flow of an SFC does not redundantly pass through the same path, thereby avoiding redundant propagation delays. Constraints () and () are binary-variable constraint conditions for α and β, respectively. The objective function (9) represents a mixed-integer nonlinear programming problem that is NP-hard. Therefore, this paper proposes an online heuristic algorithm that aims to provide a solution close to the optimal, taking into account the NP-hard nature of the problem.

### 3.2. Minimum Hop Region Formulation

The first step in solving this problem is to select valid and invalid nodes to form the rendezvous points of the routing path to reduce the computational complexity of forming routing paths for servicing the SFCs. Valid rendezvous points refer to the nodes that do not cause redundant propagation delays between the source and the destination. This subsection introduces the concept of the MHR in Walker-delta and presents techniques for setting the area of the MHR to define the candidate nodes that can serve as rendezvous points.

**Figure 2:**
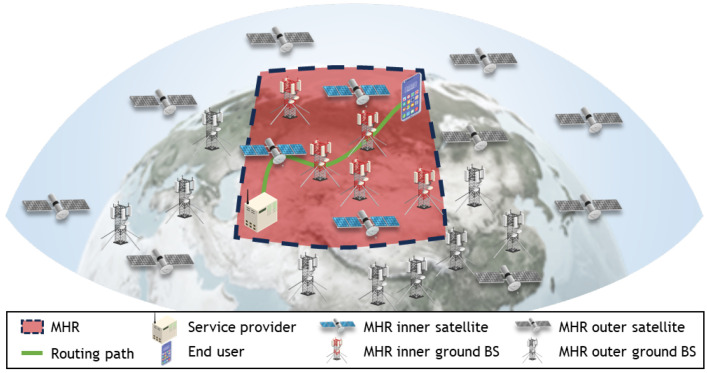
Minimum hop region for Walker-delta.

Figure 2 illustrates the MHR for Walker-Delta. The MHR represents the area in the satellite network where a rational routing path can be formed between the source and destination. Specifically, *“rational”* means that physically redundant paths are not created when forming a routing path between the source and destination within the MHR. The conventional concept of MHR was presented for the Walker-star constellation [42]. The MHR was formed in a rectangular shape in a grid-based topology for the Walker-star. This is because, in Walker-star, there exists a SEAM area where adjacent orbits move in opposite directions, preventing the formation of ISLs. However, the inclination of the orbits became relatively parallel to the equator in the Walker-delta, eliminating the SEAM area. The technique for forming an MHR in Walker-star cannot be directly applied because a grid-based topology cannot be formed in the Walker-delta. Therefore, a new technique is required to define the MHR in the Walker-delta constellation.

The MHR is determined based on the Great-circle path between the source and destination in the Walker-delta. Specifically, the MHR is determined based on the latitude and longitude of the endpoints of the Great-circle path. We checked whether both the source and destination are located in the eastern or western hemispheres longitudinally. It is assumed that negative values represent longitudes in the western hemisphere, whereas positive values represent longitudes in the eastern hemisphere. If both the source and destination are located in either the eastern or western hemispheres, then the longitudinal endpoints of the MHR are directly determined by the longitudes of the source and destination. For example, if the longitude of the source node is $30^{\circ }$ east and the longitude of the destination node is $60^{\circ }$ east, then the longitude of the MHR is $\left[30^{\circ },60^{\circ }\right]$. If one source and destination are in the eastern hemisphere and the other are in the western hemisphere, the absolute values of their longitudes are examined. If the sum of the absolute values of the longitudes of the source and destination is less than or equal to $180^{\circ }$, the Great-circle path between the two points passes through the longitude of $0^{\circ }$. In this case, the longitudinal endpoints of the MHR are set to the longitudes of the source and destination, as described earlier. Conversely, if the sum of the absolute values of the longitudes of the source and destination is greater than $180^{\circ }$, the Great-circle path between the two points passes through the longitude of $-180^{\circ }$. In this case, the longitude of the MHR is defined to include the longitudes of the source and destination nodes up to $-180^{\circ }$. By combining the four cases described above, the longitude range, $\theta_{MHR}$, of the MHR can be defined as follows:

(14) $$\begin{array}{c} \theta_{MHR}=\left\{\begin{array}{c} \left[\min\left(\theta_{s},\theta_{d}\right),\max\left(\theta_{s},\theta_{d}\right)\right]\;\mathrm{if}\;|\theta_{s}\left|+\right|\theta_{d}|\leq 180^{\circ }\;\mathrm{or}\;\theta_{s}\cdot \theta_{d}\geq 0^{\circ }\\ \left[\min\left(\theta_{s},\theta_{d}\right),-180^{\circ }\right)+\left[\max\left(\theta_{s},\theta_{d}\right),-180^{\circ }\right]\;\mathrm{if}\;|\theta_{s}\left|+\right|\theta_{d}|>180^{\circ }\; \end{array}\right. \end{array}$$

where $\theta_{s}$ represents the longitude of the source node and $\theta_{d}$ represents the longitude of the destination node.

In contrast to $\theta_{MHR}$, which does not exceed the range between the longitudes of the source and destination, the latitude of the MHR $\lambda_{MHR}$ may extend beyond the latitude range of the routing path endpoints. Owing to the characteristics of the Earth, moving the same longitude results in a shorter distance at higher latitudes than at lower latitudes. Therefore, $\lambda_{MHR}$ is determined along the Great-circle path. Fortunately, it is not necessary to check the latitude of every point along the path because the Great-circle path is always either convex or concave. First, the latitude with the largest absolute value between the latitudes of the source and destination is determined. Note that we assume positive values for north latitude and negative values for south latitude. If both the source and destination are located in the northern hemisphere or the northern node is at a higher latitude, it is necessary to check how close the Great-circle path is to the north pole. Conversely, if both the source and destination are located in the southern hemisphere, or if the southern node is at a higher latitude, it is necessary to check how close the Great-circle path is to the south pole. The latitude range of the MHR is determined by iteratively specifying points along the Great-circle path, starting from the node at a higher latitude between the source and destination and checking the latitude and longitude. For convenience, we assumed that the source node is located at a higher latitude than the destination node. Additionally, we define the latitude and longitude of the source node as $\left(\lambda_{0},\theta_{0}\right)$. The bearing angle between the source and destination nodes, $\psi_{0}$, is calculated before determining the latitude and longitude of the first exploration point. If we denote the latitude of the destination node as $\lambda_{d}$, the bearing angle to the destination node at the exploration point *x*, denoted as $\psi_{x}$, can be calculated as follows:

(15) $$\begin{array}{cc} \psi_{x}= & \arctan2\left\{\sin\frac{\theta_{d}-\theta_{x}}{2}\cdot \cos\theta_{d},\;\cos\lambda_{x}\cdot \sin\lambda_{d}-\sin\lambda_{x}\cdot \cos\lambda_{d}\cdot \cos\frac{\theta_{d}-\theta_{x}}{2}\right\}\; \end{array}$$

After computing $\psi_{0}$ by using (15), we must calculate the latitude and longitude of the first exploration point, denoted as $\left(\lambda_{1},\theta_{1}\right)$. If we have already obtained $\left(\lambda_{x-1},\theta_{x-1},\psi_{x-1}\right)$ for the (x−1)th point, we can calculate the latitude of the *x*-th point as follows:

(16) $$\begin{array}{cc} \lambda_{x}= & \arcsin\left\{\sin\lambda_{x-1}\cdot \cos\left(r_{step}\right)\cdot \cos\lambda_{x-1}\cdot \sin\left(r_{step}\right)\cdot \cos\psi_{x-1}\right\}\; \end{array}$$

where $r_{step}$ represents the distance between the (x−1)-th and *x*-th exploration points; Naturally, setting $r_{step}$ will expedite convergence but reduce the accuracy of $\lambda_{MHR}$, whereas setting $r_{step}$ smaller will have the opposite effect. Finally, the longitude $\theta_{x}$ of the *x*-th point can be calculated as follows:

(17) $$\begin{array}{cc} \theta_{x}= & \arctan2\left\{\sin\psi_{x-1}\cdot \sin\left(r_{step}\right)\cdot \cos\lambda_{x-1},\;\cos\left(r_{step}\right)-\sin\lambda_{x-1}\cdot \lambda_{x}\right\}+\theta_{x-1}\; \end{array}$$

where $\lambda_{s}$ represents the latitude of the source node. The process of determining the bearing angle, latitude, and longitude at each point is repeated iteratively by using Equations (15)–(17).

As described earlier, the iteration process can be stopped when $\lambda_{x}$ begins to decrease below $\lambda_{x-1}$ if the node with a higher latitude is located in the northern hemisphere because the Great-circle path is always either convex or concave. More specifically, if a node with a higher latitude is located in the northern hemisphere, the iteration can be halted when $\lambda_{x}$ becomes smaller than $\lambda_{x-1}$, and $\lambda_{x}$ can be set as the endpoint of $\lambda_{MHR}$. Conversely, if a node with a higher latitude is located in the southern hemisphere, the iteration can be stopped when $\lambda_{x}$ becomes greater than $\lambda_{x-1}$. In addition, a polar region boundary exists for the Walker-delta. Therefore, if the absolute value of $\lambda_{x}$ exceeds the polar region boundary, the endpoint of $\lambda_{MHR}$ is set as the latitude of the polar region boundary, denoted by $\lambda_{polar}$. Combining these explanations, the latitude range of the MHR, $\lambda_{MHR}$, can be defined as follows when the iteration ends at $\lambda_{x+1}$:

(18) $$\begin{array}{c} \lambda_{MHR}=\left\{\begin{array}{c} \left[\min\left(\lambda_{s},\lambda_{d}\right),\lambda_{x}\right]\;\mathrm{if}\;\max\left(\lambda_{s},\lambda_{d}\right)+\min\left(\lambda_{s},\lambda_{d}\right)\geq 0^{\circ }\\ \left[\lambda_{x},\max\left(\lambda_{s},\lambda_{d}\right)\right]\;\mathrm{if}\;\max\left(\lambda_{s},\lambda_{d}\right)+\min\left(\lambda_{s},\lambda_{d}\right)<0^{\circ }\\ \left[\min\left(\lambda_{s},\lambda_{d}\right),\lambda_{polar}\right]\;\mathrm{if}\;\lambda_{x+1}\geq \lambda_{polar}\\ \left[\lambda_{polar},\max\left(\lambda_{s},\lambda_{d}\right)\right]\;\mathrm{if}\;\lambda_{x+1}<\lambda_{polar} \end{array}\right. \end{array}$$

We can determine the ranges of latitudes and longitudes for the MHR, denoted by $\left(\lambda_{MHR},\theta_{MHR}\right)$. By defining the MHR area, we can avoid generating redundant propagation delays in the Walker-delta and distinguish candidate base stations that can be considered as rendezvous points. Algorithm 1 presents the pseudocode that illustrates the process of determining $\left(\lambda_{MHR},\theta_{MHR}\right)$. Specifically, each condition in (18) directly matches the corresponding steps in Algorithm 1. The first condition in (18) is handled in line 28 of Algorithm 1. The second condition in (18) is addressed in line 25. The third condition corresponds to line 20. Finally, the fourth condition is reflected in line 18.
**Algorithm 1:**Minimum Hop Region Determination 
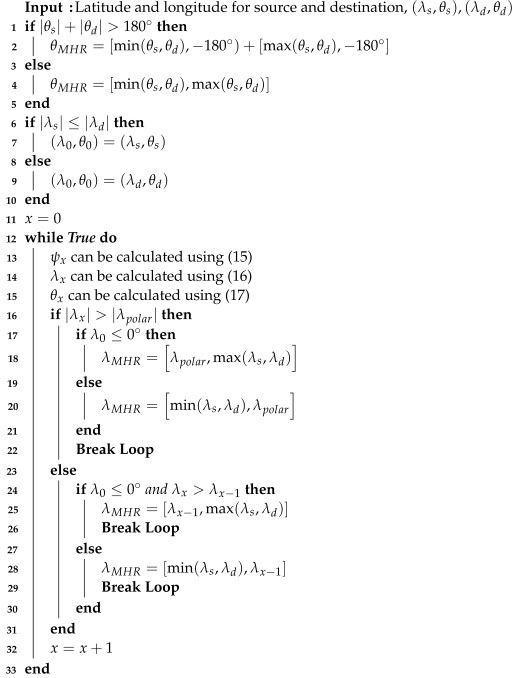


### 3.3. VNF Placement and Routing Path Formulation

The MHR formation process described in the previous subsection can be performed by setting the source to the location and destination of the service provider at the end user’s location. LEO satellites and ground base stations within the formed MHR area do not introduce redundant propagation delay, making them rational router candidates. Once candidate base stations are selected, the service provider must minimize E2E delay, form routing paths, and deploy VNFs to nodes along the path to process SFCs for end users. This subsection explains the process of forming routing paths and deploying VNFs for the service provider to process the SFCs by minimizing the E2E delay.

If the service provider decides to install SFCs that have not had their VNFs installed at any base station, it must formulate the physical routing paths before installing them. When forming routing paths, the service provider should consider the following fact: *“Transmission and computing delay can be adjusted based on how VNF installation is performed, but propagation delay remains unchanged once the physical routing path is determined”.* Therefore, the service provider formulates a routing path to minimize E2E propagation delay. Specifically, the service provider sets the propagation delay of the links between base stations, denoted by $l_{x,y}\cdot d_{l_{x,y}}^{p}$, as the weights of the communication links. The service provider then forms several routing paths using Yen’s algorithm to minimize the sum of the weights by excluding communication links with zero weights. If multiple routing paths that can minimize the propagation delay are found using Yen’s algorithm, the VNF placement algorithm, described later, is executed for each path. Naturally, there exists a tradeoff in that finding many routing paths results in a performance that approaches the global optimum at the expense of increased computational complexity.

Once the candidate nodes for VNF installation have been determined, it is necessary to decide how to deploy the VNFs. For instance, all the VNFs could be installed at the first base station on the routing path (the base station closest to the service provider). Alternatively, the VNFs could be evenly distributed among the nodes along the routing path. Given the routing path, computing and bandwidth resources of the nodes, and computing and data rate requirements of the VNFs, the simplest method to minimize the E2E delay is to explore all possible VNF deployment scenarios and perform an exhaustive search for the E2E delay. However, this exhaustive search process is computationally and temporally intensive, making it difficult to quickly determine the optimal VNF deployment scenario that minimizes the E2E delay. A VNF placement algorithm that can approximate the global optimum without exhaustively searching all scenarios is required to expedite VNF deployment.

The service provider must understand the impact on the E2E delay when VNFs are deployed relatively forward (closer to the service provider) and backward (closer to the end user) within the routing path to deploy VNFs without an exhaustive search. The service provider defines the following rule for VNF deployment to prevent redundant propagation delay: *“If the y-th VNF is installed at the x-th base station, subsequent VNFs can only be installed at nodes located later than the x-th base station”.* For example, if the second VNF is installed at the third base station along the routing path, subsequent VNFs, such as the third and fourth, can only be installed at the third, fourth, fifth, and subsequent base stations. If the second VNF is installed at the third base station and the third VNF is installed at the second base station, redundant propagation delays would occur because the link between the third and second base stations is used multiple times.

The service provider then determines the sequential installation from the first VNF. Assuming that the first VNF is installed at the *x*-th base station, the total sum of the transmission delays from the service provider to the *x*-th base station must be calculated. Specifically, the total sum of transmission delay is $\frac{w_{0}}{W_{l_{0,1}}}+\frac{w_{0}}{W_{l_{1,2}}}+\ldots +\frac{w_{0}}{W_{l_{x-1,x}}}$ considering the communication link between the *x*-th and $x^{\prime }$-th base stations along the routing path as $l_{x,x^{\prime }}$. Here, $w_{0}$ represents the data rate requirement before the first VNF is processed, and $l_{0,1}$ denotes the communication link between the service provider and the first base station. Generalizing this by assuming that the (y−1)-th VNF $f_{y-1}$ is installed at the $x^{\prime }$-th base station, the transmission delay ${\hat{d}}_{x^{\prime },x}^{t}\left(f_{y-1},f_{y}\right)$ required to install the *y*-th VNF $f_{y}$, at the *x*-th base station is as follows:

(19) $$\begin{array}{c} {\hat{d}}_{x^{\prime },x}^{t}\left(f_{y-1},f_{y}\right)=\sum_{i=x^{\prime }}^{x-1}\left(\frac{w_{f_{y-1}}}{W_{l_{i,i+1}}}\right) \end{array}$$

Equation (19) naturally increases when the VNFs are placed further along the routing path. In other words, if the service provider wants to minimize (19) to install the VNFs, there is a tendency to be installed toward the front of the routing path. If the data rate requirements of the VNFs after the *y*-th VNF are low, it may be appropriate to place VNF $f_{y}$ toward the front. However, if the data rate requirements of the VNFs after the *y*-th VNF are higher than $w_{f_{y}}$, it may be advantageous to place VNF $f_{y}$ toward the back of the routing path and overlap multiple VNF installations at the base stations placed at the back of the path. In summary, the impact of the transmission delay caused by VNFs after $f_{y}$ must be considered when installing $f_{y}$ at the *x*-th base station. Unfortunately, the exact locations where the remaining VNFs will be installed cannot be known, unlike in (19), making it impossible to accurately predict the transmission delay. Moreover, it is unsuitable for calculating all possible cases in which VNFs after $f_{y}$ can be installed at base stations beyond the *x*-th base station.

Algorithm 2 presents the pseudocode for estimating the transmission delay caused by subsequent VNFs when installing VNF $f_{y}$ at the *x*-th base station.
**Algorithm 2:**Estimation for Potential Transmission Delay 
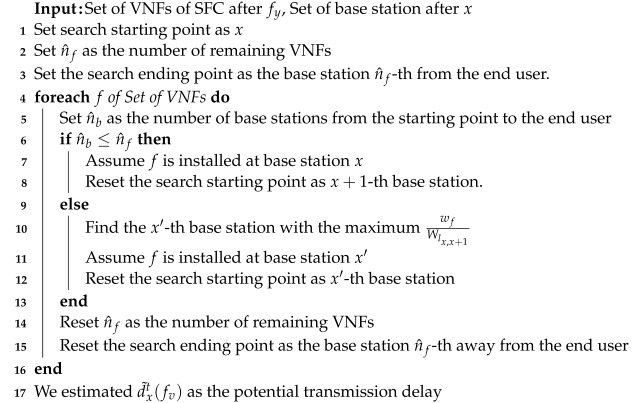


   The potential transmission delay is estimated greedily. In addition, the algorithm predicts the worst-case transmission delay that can occur when VNF $f_{y}$ is installed at the *x*-th base station. The aim is to encourage the placement of VNFs on lower-latency base stations by predicting the potential worst-case transmission delays. All the VNFs must be installed at different base stations to calculate the worst-case transmission delay. If the number of remaining base stations is less than the remaining number of VNFs, the remaining VNFs are sequentially assumed to be installed, starting at the base station of the search. Otherwise, the algorithm assumes the installation of the VNF at the base station, which causes the maximum transmission delay. Once these VNF placement scenarios are established, the algorithm estimates the maximum potential transmission delay ${\tilde{d}}_{x}^{t}\left(f_{v}\right)$. ${\tilde{d}}_{x}^{t}\left(f_{v}\right)$ obtained using Algorithm 2 tends to decrease as the VNFs are placed further back along the routing path.

Equation (19) encourages the service provider to consider installing the *y*-th VNF in the front of the routing path. Conversely, Algorithm 2 encourages the placement of the *y*-th VNF toward the rear of the routing path. Therefore, the service provider computes ${\hat{d}}_{x^{\prime },x}^{t}\left(f_{y-1},f_{y}\right)$ and ${\tilde{d}}_{x}^{t}\left(f_{v}\right)$ for each possible base station *x* when deciding where to install the *y*-th VNF. The sum of these two transmission delays is considered as the effect of installing the *y*-th VNF at the *x*-th base station.

The impact of the computing delay when installing the *y*-th VNF at the *x*-th base station must be considered, similar to the transmission delay. The most naive approach is to install a VNF on the base station with the highest computing power $P_{x}$. However, two points must be considered. The first point to consider is that if a base station with high computing power is located at the rear of the routing path, the remaining VNFs may be excessively overlapped with the VNFs. When a base station installs multiple VNFs simultaneously, its computing power is evenly distributed among the VNFs. Therefore, excessive overlapping of VNFs may amplify the computing delay. Thus, the computing delay when installing the *y*-th VNF at the *x*-th base station is calculated by assuming that the remaining VNFs are evenly distributed among the remaining routers and then amplifying the computing requirements $p_{f}$ of the VNF $f_{y}$. Similarly, the second point to consider is that if the *y*-th VNF is additionally installed at the base station *x*, which already has $n^{\prime }$ VNFs installed, the computing delay should be calculated as $\frac{\left(n^{\prime }+1\right)\cdot p_{y}}{P_{x}}$. The service provider calculates the impact of the computing delay when installing the *y*-th VNF at the *x*-th base station using the aforementioned method.

The service provider calculates the sum of the computing and transmission delays incurred when installing VNF $f_{y}$ at each base station along the routing path. The service provider then installs VNF $f_{y}$ at the base station with the lowest total delay sum. This process is repeated for the (y+1)-th VNF. After installing the final VNF, adjustments are made to the values of $d_{l_{x,x^{\prime }}}^{p}$ for the subsequent SFC installation. Specifically, the computing and transmission delays that are unavoidably caused by the installed SFC are considered as propagation delays that guide the subsequent SFC to avoid the corresponding base station and routing path. The transmission delay $d_{l_{x,x+1}}^{t}\left(f_{y}\right)$ incurred on communication link $l_{x,x+1}$ owing to the installation of VNF $f_{y}$ is added directly to $d_{l_{x,x+1}}^{p}$. The computing delay $d_{x}^{c}\left(f_{y}\right)$ incurred at base station *x* owing to the installation of VNF $f_{y}$ is added to all $d_{l_{x,x^{\prime }}}^{p}$ associated with base station *x*. Subsequently, the service provider considers the updated propagation delay when forming a new routing path to serve a new SFC. Algorithm 3 represents the pseudocode for the aforementioned routing path formation and VNF placement algorithm.
**Algorithm 3:**Joint Routing Path Formulation and VNF Placement 
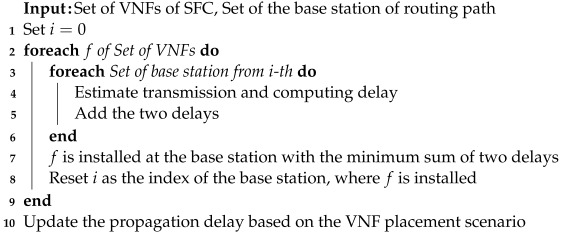


## 4. Simulation Results

In this section, we evaluate the performance of the joint routing path and VNF placement algorithm proposed in this paper. Here, we describe the simulator environment used in our experiments. We evaluated the performance by varying these three parameters. The first parameter is the Great-circle distance between the service provider and the end user. The second parameter involves changing the number of SFCs provided by the service provider and the number of VNFs composing the SFC. The third parameter involves changing the number of LEO satellites and ground base stations composing the SGIN.

### 4.1. Simulation Environment

We implemented three benchmark schemes to compare the performance of the proposed scheme. The specific descriptions of each scheme are as follows:

- *Exhaustive search* (ES): In this scheme, the service provider considers all possible routing paths within MHR and VNF placement cases in the SGIN to find the one that minimizes the E2E delay. This scheme serves as a reference to evaluate how closely the proposed scheme approaches the global optimum, despite its high computational and time complexity.
- *Proposed scheme* (PR): In this scheme, the service provider identifies candidate base stations using Algorithm 1 and places VNFs using Algorithm 3 within the three shortest routing paths by using Yen’s algorithm.
- *Greedy approach* (GR): In this scheme, the service provider minimizes the computational complexity by using the Dijkstra algorithm to form routing paths with minimal propagation delay. Subsequently, VNFs are placed on the base station with the lowest combined computing and transmission delay along the formed routing path. For example, if the delay incurred for processing the first VNF is minimal at the second base station along the path, the first VNF is installed at the second base station.

Table 1 lists the system parameters used in the simulations. The system parameters are configured based on [33,34,43]. It is assumed that the service provider is located in Seoul, and the end user is randomly positioned at a specified Great-circle distance from Seoul. All the performance values displayed in the graphs were determined as the average of 10,000 experiments, varying the resource requirements of the VNFs, the locations of the base stations constituting the SGIN, and the computing and communication resources of the base stations.

### 4.2. Impacts of Great-Circle Distance

**Figure 3:**
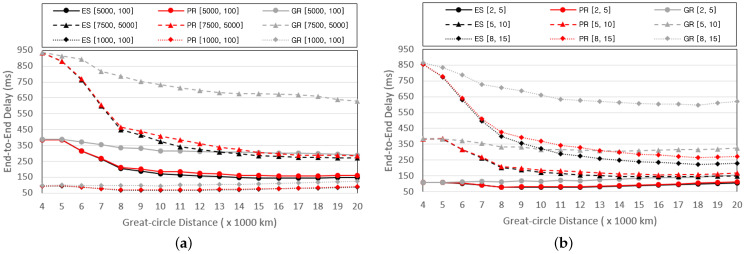
E2E delay based on the Great-circle distance and (**a**) $p_{f}$ and $w_{f}$, (**b**) $n_{c}$ and $n_{f}$.

We compared the performance of the three schemes by varying the Great-circle distance between the service provider and the end user. The Great-circle distance is within the range of $r_{th}$ to 20,000 km with intervals of 1000 km. Figure 3 presents graphs depicting the variation in the E2E delay with respect to the Great-circle distance. Figure 3a measures the E2E delay while changing the computing capacity of the VNFs, $p_{f}$, and the size of the data after processing, $w_{f}$. The values of $p_{f}$ and $w_{f}$ for the VNFs are randomly determined within predefined ranges using a uniform distribution. Figure 3b measures the E2E delay while varying the number of SFCs and the number of VNFs composing each SFC. We represent the scenarios with *x* SFCs and *y* VNFs per SFC as [x,y].

In Figure 3a, it can be observed that, regardless of the scheme type, the E2E delay increases as the distribution of computing and communication resources required to process the VNFs increases. This is because the computing and transmission delays incurred in processing and transmitting the VNFs increase proportionally with the resource requirements. Another notable finding is that despite the increase in the Great-circle distance, a general trend of decreasing E2E delay exists. This can be attributed to the increase in the number of base stations included in the MHR as the Great-circle distance increases. The number of base stations included in the routing path increases with the number of base stations included in the MHR. The VNFs can be distributed across multiple base stations as the number of base stations that form a routing path increases. Consequently, the computing delay decreases as the Great-circle distance increases, resulting in a reduction in the E2E delay. However, a different phenomenon occurs when the Great-circle distance is greater than 10,000 km in the [1000,100] scenario. This is because the computing power of the base stations in the routing path is already sufficient, causing an increase in the propagation delay to outweigh the decrease in the computing delay. Consequently, the E2E delay increases slightly. The higher E2E delay observed in PR compared to ES can be attributed to the discrepancy between the estimated transmission delay calculated using Algorithm 2 and the actual transmission delay. Despite this discrepancy, the similarity in E2E delay between PR and ES indicates that Algorithm 2 effectively predicts the potential transmission delay. Moreover, the PR can quickly approximate values close to the optimum with a complexity of $O(n_{m,f})$, whereas the ES exhaustively searches for all possibilities. This demonstrates that while PR may have a slightly higher E2E delay, its efficiency in approximating the optimum solution makes it a practical alternative to ES. For PR, when [1000,100], it shows 97% similarities to the E2E delay of ES. In this case, it implies that the computing requirement of VNF is low, and the resulting data size after processing is also small. Thus, the proportion of computing and transmission delay becomes minimal, making the impact of propagation delay more significant. As a result, placing the VNF on the shortest physical path becomes close to the optimal solution in most cases, leading to the observed 97% similarity. In other general experimental results, we observed that PR, on average, shows 90% similarity to the E2E delay of ES. The GR scheme exhibits the highest E2E delay when the base station suitable for installing VNFs is located at the rear of the routing path, resulting in the duplication of all VNFs in the rear. In Figure 3b, it is also observed that as the number of SFCs and VNFs increases, the computing and transmission delay increases, increasing the E2E delay. In both graphs, the difference in E2E delay between the schemes is not significant when the Great-circle distance is small. This is because fewer base stations exist within the MHR, resulting in fewer routing paths and fewer VNF installation cases.

**Figure 4:**
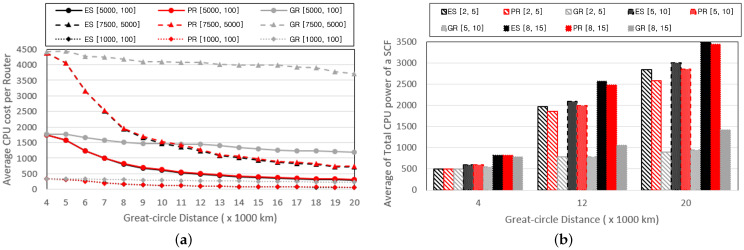
Performance changes of (**a**) average CPU cost per router and (**b**) total CPU power to process an SFC based on the Great-circle distance.

Figure 4 depicts graphs showing the variation in the CPU cost with respect to the Great-circle distance. The CPU cost of a single base station is defined as the product of the number of installed VNFs and the sum of $p_{f}$ values of the VNFs. Figure 4a illustrates the graph that shows the average CPU costs assigned to a single base station. Figure 4b illustrates the graph representing the averages of the total CPU power of the base stations serving SFCs. Importantly, base stations without installed VNFs are not included in the averages. Figure 4a exhibits a trend similar to that shown in Figure 3a. This is because the CPU cost assigned to the base stations has an immediate impact on computing delay. As the MHR increases proportionally with the Great-circle distance, the number of base stations that form the routing path also increases, leading to the distribution of VNFs across multiple base stations and subsequently reducing the CPU cost of the base stations. Similarly, the total CPU power of the base stations that serve the SFCs increases proportionally with the Great-circle distance, as shown in Figure 4b. Additionally, Figure 4b shows that the total CPU power increases proportionally with the number of SFCs and VNFs. This is because, as the number of VNFs increases, the number of base stations installing VNFs to process the SFCs also increases. The PR scheme exhibits a slightly higher CPU cost and slightly lower CPU power than the ES, which examines all routing paths. In the case of the GR scheme, the occurrence of duplicating multiple VNFs in the rear of the routing path increases the CPU cost and decreases the total CPU power.

### 4.3. Impacts of Configuration of SFCs

**Figure 5:**
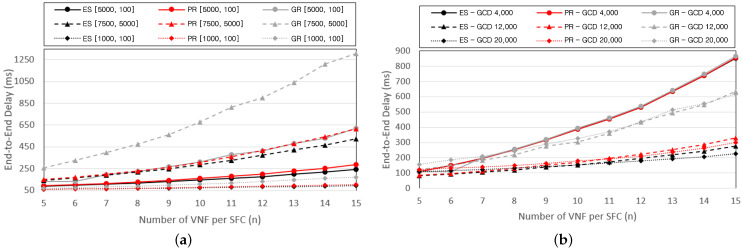
E2E delay based on the number of VNFs per SFC and (**a**) $p_{f}$ and $w_{f}$; (**b**) Great-circle distance.

We compare the performance by varying the number of SFCs provided by the service provider and the number of VNFs composing each SFC; the number of VNFs varies from five to fifteen, and the number of SFCs varies from two to eight. Figure 5 depicts graphs of the E2E delay with changes in the number of VNFs composing the SFCs. Figure 5a illustrates the change in E2E delay while varying the range of resource requirements for the VNFs, with five SFCs and a Great-circle distance of 15,000 km. Figure 5b represents the change in the E2E delay while varying the Great-circle distance between the service provider and end user, with the range of resource requirements for VNFs set to U[5000,100]. Similar to the reasons in Figure 3a and Figure 4a, as the resource requirements of the VNFs increase, the computing and transmission delays also increase, increasing the E2E delay. Additionally, an increase in the number of VNFs results in a proportional increase in the E2E delay. Unlike the GR scheme, the PR scheme exhibits a relatively gradual increase in E2E delay with an increase in the number of VNFs because it prevents the duplication of VNF installations in the rear part of the routing path using Algorithm 2. Therefore, it exhibits E2E delays that are relatively similar to those of the ES. All three schemes exhibit similar performances in the case of a Great-circle distance of 4000 km, where the number of base stations in the MHR area is small. However, the PR scheme drastically reduces the E2E delay, similar to the ES scheme, as the MHR area increases, whereas the GR scheme shows a relatively marginal decrease in the E2E delay owing to the clustering of VNFs in the rear part of the routing path.

**Figure 6:**
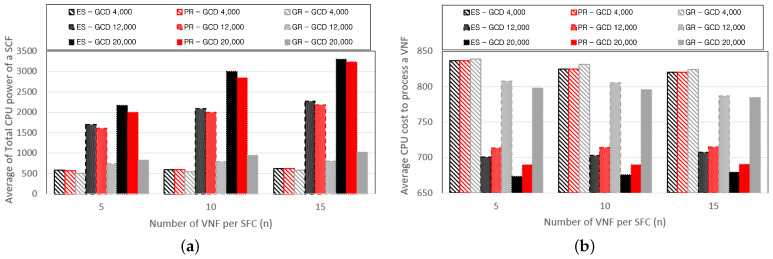
Performance changes of (**a**) the total CPU power of an SFC, and (**b**) the average CPU cost to process a VNF according to the number of VNFs per SFC.

Figure 6 depicts graphs showing the performance related to the computing power while varying the Great-circle distance. Figure 6a represents the average total CPU power of the base stations deployed to process the VNFs composing the SFCs. Base stations without installed VNFs are not included in the computations. Figure 6b illustrates the average CPU cost incurred to process a single VNF, where the CPU cost for processing VNFs is defined as the quotient of the CPU cost per base station and the number of VNFs installed. The total CPU power required to service the SFCs increases with the number of VNFs and the length of the Great-circle distance. With a larger number of VNFs, the schemes tend to be distributed across multiple base stations, resulting in an increase in CPU power. Similarly, an increase in the Great-circle distance induces the dispersion of VNFs across multiple base stations, increasing the CPU power. PR prevents the excessive duplication of VNF installations on the same base station through Algorithm 3, thereby ensuring higher CPU power compared with GR. As the Great-circle distance increases, the VNFs are distributed across multiple base stations, decreasing the CPU cost required to process a single VNF. However, it can be observed that the CPU cost incurred for VNF processing is not significantly affected by the number of VNFs composing the SFCs. This is because the PR scheme determines the distribution of VNFs across multiple base stations based on the CPU cost using Algorithm 3. The PR scheme tends to install additional VNFs on base stations with lower CPU costs while avoiding additional installations on base stations with higher CPU costs, resulting in similar CPU costs regardless of the number of VNFs.

**Figure 7:**
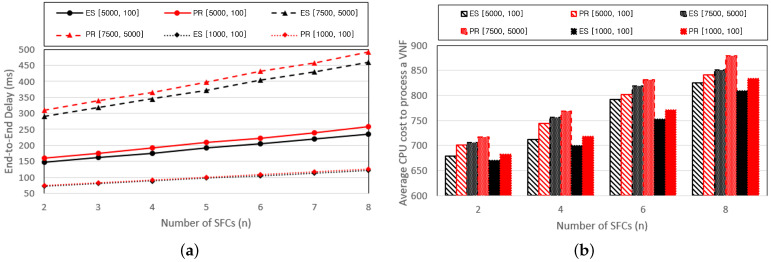
Performance changes of (**a**) E2E delay and the (**b**) average CPU cost based on the number of VNFs per SFC.

Figure 7 illustrates graphs that compare the performance while varying the number of SFCs provided by the service provider. The number of VNFs composing the SFC was fixed at ten. Figure 7a presents graphs that compare the E2E delay with changes in the distribution of the VNF resource requirements. Figure 7b represents a graph that depicts the CPU cost incurred to process a single VNF. There exists a tendency for both the E2E delay and CPU costs to increase proportionally with an increase in the number of SFCs. This is attributed to the increase in the total number of VNFs installed on the SGIN as the number of SFCs increases, increasing the computing and transmission delays.

### 4.4. Impacts of the Number of Base Station Nodes

**Figure 8:**
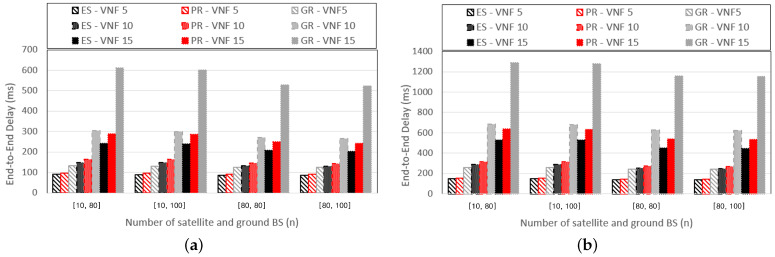
E2E delay according to the SGIN configuration with (**a**) $p_{f}=U\left[5000,100\right]$, (**b**) $p_{f}=U\left[7500,5000\right]$.

We compare the performances of the three schemes by varying the number of satellites and ground base stations in the SGIN. For convenience, we denote the number of satellites $n_{s}$ and ground base stations $n_{g}$ in the graphs as $\left[n_{s},n_{g}\right]$. Figure 8 presents graphs that compare the E2E delay while varying the SGIN configuration and the number of VNFs that compose the SFC. The number of SFCs provided is fixed at eight. Figure 8a and Figure 8b represent graphs for cases where the resource requirements of the VNFs are U[5000,100] and U[7500,5000], respectively. As the number of base stations increases, the number of base stations within the MHR also proportionally increases. Consequently, the number of base stations that compose the routing path also increases proportionally, leading to a reduction in the E2E delay owing to the decreased computing delay. In addition, there exists a higher probability of forming routing paths similar to the shortest physical distance as the number of base stations increases, leading to a decrease in propagation delay.

**Figure 9:**
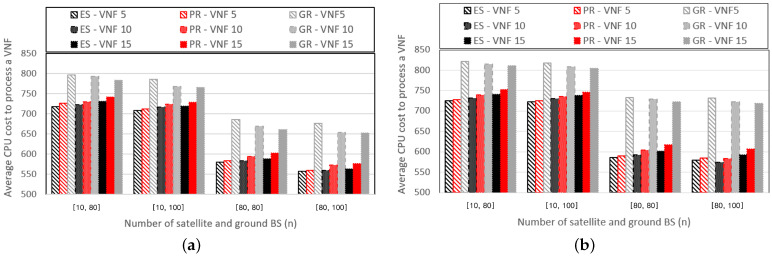
Average CPU cost to process a VNF according to the SGIN configuration with (**a**) $p_{f}=U\left[100,5000\right]$, (**b**) $p_{f}=U\left[5000,7500\right]$.

Figure 9 shows graphs that depict the CPU cost required to process a single VNF while varying the SGIN configuration. Figure 9a and Figure 9b represent graphs for cases where the resource requirements of the VNFs are U[5000,100] and U[7500,5000], respectively. Unlike the number of VNFs, it can be observed that as the number of base stations increases, the CPU cost required to process a single VNF decreases. This is because the number of base stations that form a routing path is affected by the SGIN configuration. When more base stations are included in the routing path, additional opportunities to distribute VNFs exist, decreasing the CPU cost required to process a single VNF. Furthermore, even as the number of base stations increases, the performance difference between the PR and ES schemes remains relatively consistent. However, it can be concluded that the PR scheme becomes more advantageous as the number of base stations in the SGIN increases, considering that the complexity of the ES scheme increases more rapidly than that of the PR scheme as the number of base stations increases.

### 4.5. Comparison with Other Methods

At present, there exists one research study that jointly performs VNF placement and routing path formation to minimize E2E delay [12]. The authors aim to minimize E2E service latency by jointly optimizing VNF placement and routing. To achieve this, they proposed a time-expansion-based decoupled greedy (TEDG) algorithm, which is a heuristic algorithm that aims to reduce computational complexity while achieving near-optimal performance. For the performance comparison, they introduced the following four methods:

- *TEDG with the modified max-min normalization* (MM): The MM method aims to better utilize available resources by adjusting the weights to improve the selection of routing paths and the allocation of VNFs, focusing on optimizing both latency and resource usage.
- *TEDG with equal weight* (EW): A variation of the TEDG algorithm where the cost of all available links, including communication and stay links, is assigned an equal weight of 1.
- *Decoupled greedy algorithm* (DG): A modified version of a decoupled greedy algorithm, which allocates resources to services based on a configuration period rather than a time slot.
- *Genetic algorithm* (GA): A genetic algorithm applied to solve the joint VNF placement and routing planning problem. This approach is also used in similar contexts for optimizing resource allocation in cloud centers.

We intend to compare the proposed MM, EW, DG, and GA algorithms from the referenced paper alongside the ES and PR algorithms from our experiments to demonstrate the superior performance of our approach. The experimental environment and parameters were the same as those used in the work.

**Figure 10:**
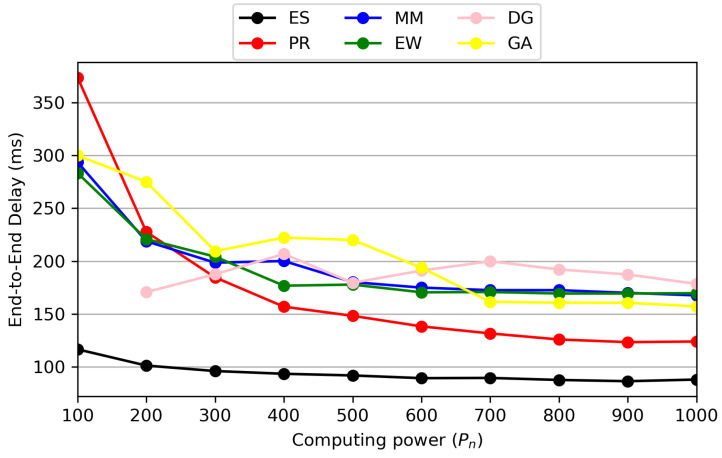
Performance comparison of E2E delay based on computing power.

Figure 10 is a performance analysis graph of end-to-end delay as a function of computing power. As shown, the ES algorithm consistently achieves the lowest E2E delay since it finds the optimal solution. In the case of the PR algorithm, it begins to outperform other methods starting from 300 units of computing power. The proposed PR method places VNFs based on the predicted transmission delay, which is calculated using computing power divided by transmission data rates. When computing power is low, the prediction accuracy of our method decreases, resulting in significant performance degradation compared to ES. However, as computing power increases, the accuracy improves, allowing the PR method to achieve performance similar to that of the ES method.

One drawback of the proposed method is that E2E delay significantly increases in scenarios with low computing power. However, in 3GPP, there are plans to provide regenerative payload services utilizing LEO satellite networks [44]. In other words, by using LEO satellites as base stations, it is possible to deploy VNFs for service provisioning. Based on this development, it is expected that each satellite network will have sufficient computing power, though perhaps not as much as ground stations. Therefore, we believe that our proposed method can outperform other existing techniques in real-world scenarios.

### 4.6. Analysis of the Scalability

To demonstrate the scalability of our method, we conducted experiments focusing on three aspects: (i) performance comparison based on the number of users, (ii) performance comparison based on the number of SFCs, and (iii) performance comparison based on the number of VNFs. To verify the scalability of our method, we evaluated its performance under two scenarios: our main scenario and a highly loaded scenario. The parameters for the three aspects, $n_{u}$, $n_{c}$, and $n_{f}$, were set to 10, 5, and 20, respectively, in the main scenario and 30, 20, and 40, respectively in the highly loaded scenario. Other parameters were taken from Table 1. The experiments are designed to measure the E2E delay, allowing us to assess how effectively our method scales under different network conditions and parameter settings.

**Figure 11:**
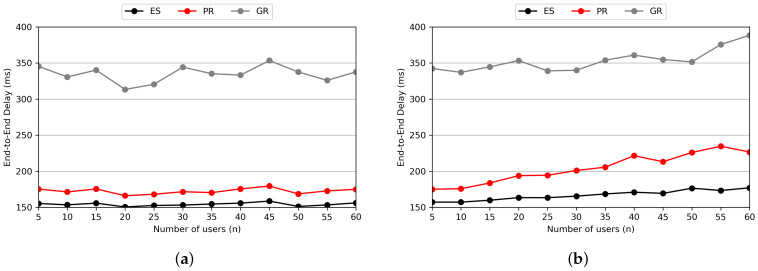
Average E2E delay according to the number of users in (**a**) main scenario and (**b**) highly loaded scenario.

Figure 11 represents the performance comparison based on the number of users. As seen in Figure 11a, within the MHR-scale region we consider, PR shows performance similar to ES even as the number of users increases. This indicates that the E2E delay does not increase significantly because there are relatively few types of SFCs, meaning that the increasing number of users leads to handling similar requests. On the other hand, in Figure 11b, where a higher number of SFCs and VNFs are considered, it is evident that as the number of users increases, the increase in E2E delay is larger compared to ES. This delay increase occurs because low-delay paths become occupied as different types of SFCs are requested.

**Figure 12:**
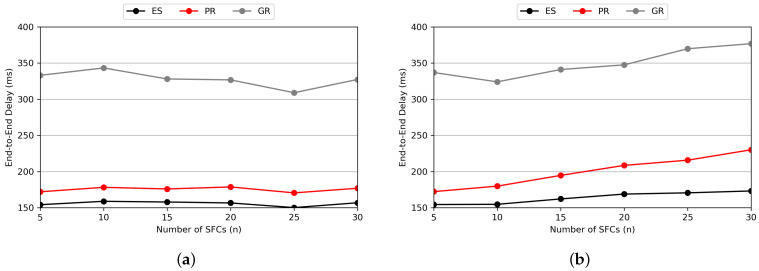
Average E2E delay according to the number of SFCs in (**a**) the main scenario and (**b**) the highly loaded scenario.

Figure 12 shows the performance comparison based on the number of SFCs. In Figure 12a, even as the variety of SFCs increases, the performance shows little change since the number of users is fixed. Therefore, we can see an increase in delay only up to the point where the number of users matches the number of SFC types, which is ten. In Figure 12b, as the number of users increases, the E2E delay continues to increase. This result is similar to that of the experiment on the number of users, indicating that there is a limitation in VNF placement as various types of SFCs are requested. However, even under a highly loaded scenario, a perfectly individual SFC request situation is unlikely to occur. Thus, we expect that such an increase in E2E delay would not happen within the MHR scenario we consider.

In terms of the number of VNFs, we conducted experiments with the number of VNFs in a single SFC randomly set within the range [5, 15] in our typical experimental setup. Therefore, examining the number of VNFs per SFC, rather than merely increasing the total number of VNFs, would better reflect scalability. This experiment has been analyzed in detail in Figure 5, Figure 6 and Figure 7.

From a scalability perspective, we confirmed that the E2E delay increased as the number of users, SFCs, and VNFs increased. Our method focuses on VNF placement and routing path formation within the MHR region. By reducing the problem scope to the MHR, the proposed method is expected to achieve the desired performance in the main scenarios we consider. In other words, we can claim that by solving the problem within the MHR, we have secured scalability for our proposed method.

### 4.7. Analysis of the Confidence Interval

Since our experiments involve a large number of simulations from various aspects and present averaged results, an analysis of how the E2E delays are distributed is necessary. To derive this distribution, we conducted 10,000 experiments using the parameters in Table 1 and represented the results as a probability density function (PDF). Then, we calculated the overall average E2E delay and derived a 95% confidence interval centered on this average to analyze the distribution of E2E delays.

**Figure 13:**
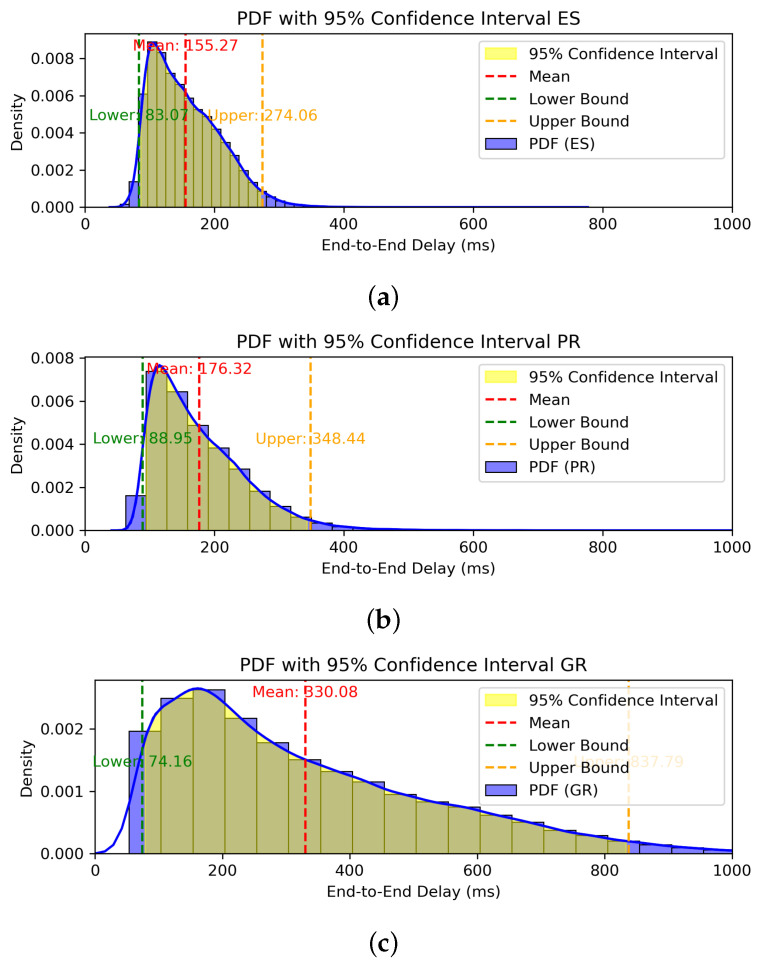
PDFs of E2E delay with 95% confidence interval of (**a**) ES, (**b**) PR, and (**c**) GR.

Figure 13 shows PDFs with a 95% confidence interval of (a) ES, (b) PR, and (c) GR. Comparing the performance of ES and PR, PR shows an increase of approximately 11.93%, 4.32%, and 26.59% in the mean, lower bound, and upper bound, respectively, compared to ES. Although the upper bound shows a substantial increase in E2E delay, most E2E delays are observed to be distributed below the mean value. Therefore, we can conclude that the proposed method exhibits performance similar to that of ES in most cases.

## 5. Conclusions

In this paper, we proposed a scheme for VNF placement and routing path formulation in SGIN, aiming to minimize E2E delay. The proposed scheme introduces an MHR method to streamline the identification of candidate base stations for routing paths, thereby reducing computational complexity. By leveraging only the latitude and longitude of the service provider and end user, the MHR effectively narrows down the range of candidate base stations to be considered. Additionally, we developed an algorithm for routing path formation and VNF placement, considering the resource requirements of VNFs, such as computing power and communication link data rates. The routing paths were designed to minimize propagation delays, and VNFs were strategically placed along the paths to reduce both computational and transmission delays. Through simulation-based experiments, we demonstrated the effectiveness of the proposed technique, achieving up to a 97% similarity in performance to the exhaustive search method, with an average similarity of approximately 90%, in terms of minimizing E2E delay while significantly lowering computational complexity. For future work, we identify the need to consider the frequency of SFC invocations. In the current scheme, initially invoked SFCs occupy the optimal routing paths, which may not be ideal when multiple end users are involved. To further optimize E2E delay, routing paths and VNF placements should be dynamically adjusted based on the frequency of SFC invocations. Future research will focus on developing mechanisms that allocate optimal routing paths to frequently invoked SFCs, ensuring efficient VNF placement and minimizing delays across multiple service instances.

## Figures and Tables

**Table 1:** Simulation parameters.

Notation	Description	Value
$n_{s}$	The number of satellites	5
$n_{u}$	The number of users	10
$n_{g}$	The number of ground base stations	80
$n_{f}$	The number of VNFs	20
$n_{c}$	The number of SFCs	[2,8]
$p_{f}$	The computing power requirement of the VNF *f*	U[100,7500]
$P_{n}$	The computing power of base station node *n*	U[250,500] (for satellite) U[500,1000] (for ground)
$W_{x,y}$	The data rate of the link	U[500,1000]
$w_{f}$	The resulting data size after processing *f*	U[100,7500]
$r_{th}$	The maximum communication distance	4000 (km)

## Data Availability

The data generated during this current study are available from the authors upon reasonable request.

## Abbreviations

The following abbreviations are used in this manuscript:

SGIN	Satellite–ground integrated network
QoS	Quality of Service
LEO	Low-Earth orbit
SDN	Software-defined networking
NFV	Network function virtualization
VNF	Virtual Network Function
E2E	End-to-end
SFC	Service Function Chain
MINLP	Mixed integer non-linear programming
MHR	Minimum hop region
